# Triple Cardiac Procedure Through Left Periareolar Incision: Coronary Artery Bypass, Mitral Valve Repair, and Ventricular Pseudoaneurysm Repair

**DOI:** 10.1016/j.atssr.2026.01.016

**Published:** 2026-02-09

**Authors:** Hemn Abdulrahman Abdullah, Darya Nadir Saeed, Jwan Ali Ahmed AlSofi, Marwan Abdullah Barzani

**Affiliations:** 1 Department of Cardiovascular and Thoracic Surgery, Arzheen Hospital, Erbil, Iraq; 2 Department of Surgery, Hawler Teaching Hospital, Erbil, Iraq

## Abstract

Minimally invasive techniques offer advantages over sternotomy for cardiac surgery. We report a triple combined procedure—coronary artery bypass grafting, mitral valve repair, and left ventricular pseudoaneurysm repair—performed through a single left periareolar incision by using an endoscopic-assisted approach in a 54-year-old man with ischemic cardiomyopathy. The operation and recovery were uneventful. Postoperative imaging confirmed competent valve function, improved ejection fraction, restored ventricular geometry, and graft patency. At 30 days, the patient showed stable cardiac function and excellent cosmetic healing. This case demonstrates the feasibility and potential benefits of extending the periareolar approach to complex cardiac procedures with favorable outcomes.

Left ventricular (LV) pseudoaneurysm, a recognized sequela of myocardial infarction, frequently coexists with ischemic mitral regurgitation and LV systolic dysfunction, all of which significantly worsen prognosis if left untreated.^1^ Surgical management often requires combined cardiac procedures, traditionally performed through full median sternotomy. In recent years, minimally invasive cardiac surgery has gained prominence for its ability to reduce surgical trauma, transfusion requirements, and recovery time without compromising safety in combined cardiac surgical procedures.^2^^,^^3^ Among these, periareolar access offers additional cosmetic and functional advantages.^4^ This report demonstrates the feasibility of extending the periareolar approach to a triple combined procedure: coronary artery bypass grafting (CABG), mitral valve repair (MVr), and LV pseudoaneurysm repair performed through endoscopically assisted left periareolar incision.

A 54-year-old man with a body mass index of 32.6 kg/m^2^ and a medical history of hypertension and diabetes presented with exertional dyspnea classified as New York Heart Association functional class III and recurrent angina consistent with Canadian Cardiovascular Society class III. He had not sought medical care until recently, although he had received a diagnosis of coronary artery disease detected by angiography and an LV aneurysm detected by echocardiography 7 months before this presentation.

He recently experienced a myocardial infarction and underwent coronary angiography, which demonstrated a critical bifurcation lesion in the midleft anterior descending (LAD) artery, a mild critical lesion in the first obtuse marginal (OM) branch, and total occlusion of the second OM branch. Baseline echocardiography revealed enlargement of the left atrium and left ventricle, along with severe LV systolic dysfunction (ejection fraction, 25%-30%). The mitral valve demonstrated severe eccentric mitral regurgitation, with an annular diameter of 37 mm. Regional assessment revealed a large inferolateral LV aneurysm. These findings raised concern for a structural complication and prompted tissue characterization and viability imaging.

Cardiac magnetic resonance imaging confirmed a severely dilated LV with multisegment hypokinesia, identifying a large anterolateral LV pseudoaneurysm measuring 71 × 47 mm, with a 2.6-cm neck and a laminated mural thrombus (Figure 1). Valve mapping reported severe mitral regurgitation (regurgitant fraction, 68%). Late gadolinium enhancement showed transmural scar along the wall of the pseudoaneurysm with associated pericardial enhancement. No late gadolinium enhancement was observed in the LAD or right coronary artery territories, thus indicating preserved myocardial viability in those regions. However, poor viability was noted in the left circumflex artery territory (possible inferolateral exception).

**Figure 1:**
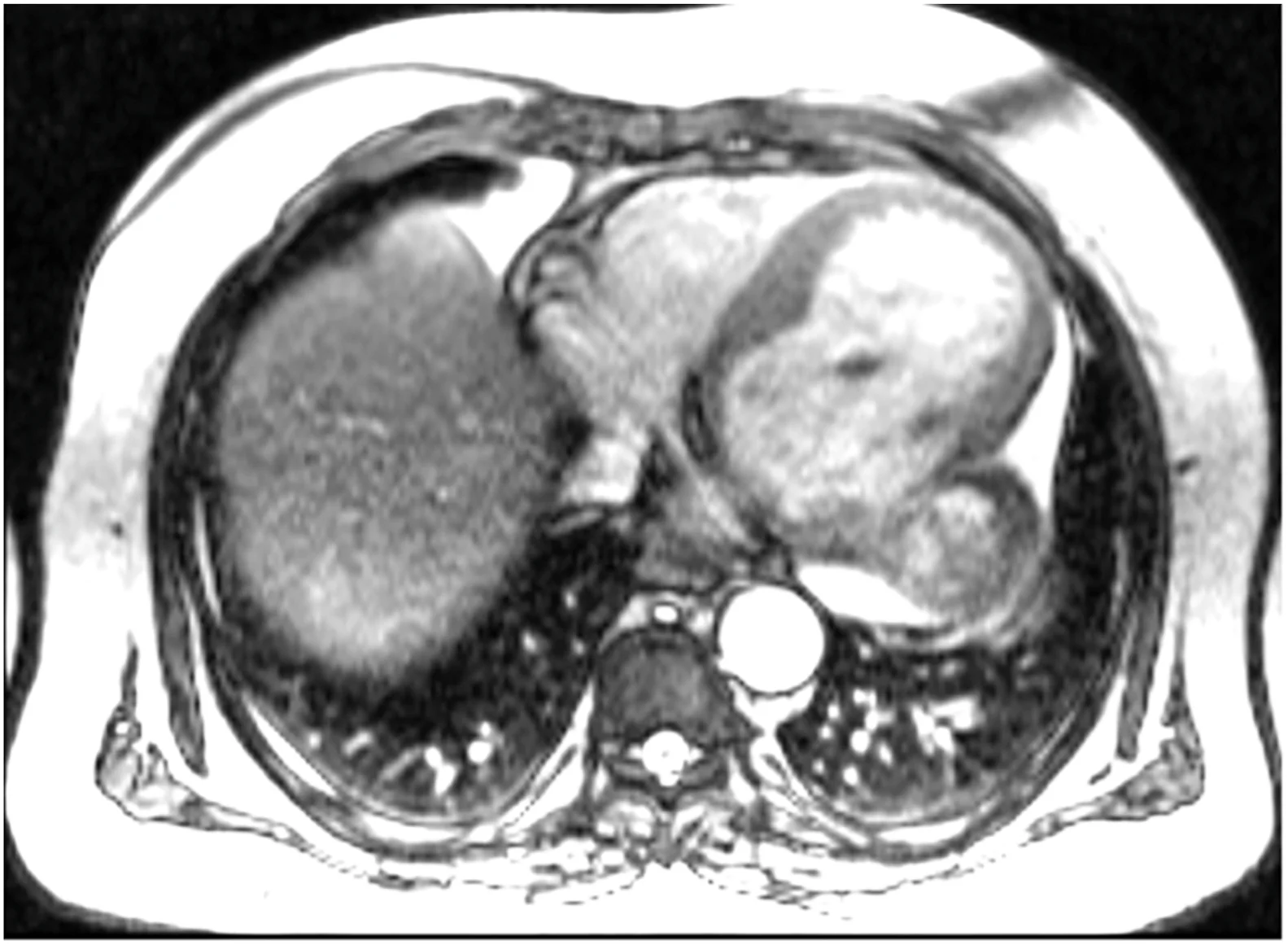
Preoperative cardiac magnetic resonance imaging showing a large left ventricular pseudoaneurysm arising from the anterolateral wall.

The patient was scheduled for surgery, with a normal range of preoperative investigations and a calculated EuroSCORE II (European System for Cardiac Operative Risk Evaluation II) of 7.8%. The Research Ethics Committee of the Dr Hemn Foundation’s research center approved the study (approval reference number 21; August 23, 2025). Informed consent was obtained from the patient before proceeding.

Under general anesthesia, the patient underwent invasive monitoring with arterial and central venous lines, along with intraoperative transesophageal echocardiography. He was positioned supine with a 15° elevation of the left shoulder. Initial surgical access was obtained through a left periareolar incision at the fourth intercostal space, with partial resection of the fourth rib. The left internal thoracic artery (LITA) was then harvested endoscopically through an additional 10-mm port placed 2 interspaces above the periareolar incision, between the anterior and midaxillary lines. Cardiopulmonary bypass was established through femoral artery and vein cannulation. On exploration using an endoscopic-assisted approach, the patient was found to have a markedly enlarged heart, with a large anterior LV aneurysm. Extensive pericardial adhesions surrounding the large LV pseudoaneurysm were encountered and were carefully lysed using scissors and monopolar electrocautery. After aortic cross-clamping with a Chitwood transthoracic clamp (Scanlan International), antegrade cardioplegia was administered. The pseudoaneurysm was opened, the thrombus was evacuated, and the resection was carried out up to the aneurysmal neck. Ischemic MVr was performed through the aneurysmal opening by using 2 Alfieri edge-to-edge sutures: 1 centrally between A2 and P2 and another at the anterolateral commissure, both secured with 5-0 Gore-Tex (W.L. Gore & Associates) U sutures. LV volume was measured using a glove filled with 120 mL of saline, and a Gore-Tex patch was fashioned to the defect size and sutured in place with continuous 3-0 polypropylene (Prolene, Ethicon) suture. The pseudoaneurysm edges were further reinforced with polytetrafluoroethylene (Teflon, Chemours) felt strips interrupted by 0 Prolene followed by the over-and-over technique with double-layer 0 Prolene. Coronary revascularization was then achieved on a beating heart using an LITA-to-LAD anastomosis and a composite LITA-radial T-graft configuration to the OM. Deairing was carefully performed in a standard fashion under transesophageal echocardiographic guidance using antegrade aortic venting through a root vent, before cross-clamp removal.

Postrepair transesophageal echocardiography confirmed competent mitral valve function and satisfactory LV geometry without residual regurgitation. The patient was weaned uneventfully from cardiopulmonary bypass with minimal inotropic support. Operative metrics included a cardiopulmonary bypass time of 220 minutes and an aortic cross-clamp time of 150 minutes. Two drains were placed: a 24F pericardial drain and a 28F pleural drain. The ribs were approximated with polyglactin 910 (Vicryl, Ethicon) sutures, muscles were reapproximated, fascia was closed with interrupted sutures, and the skin was closed subcuticularly.

He was extubated 3 hours postoperatively, experienced an uneventful 18-hour intensive care unit stay, and was discharged home in stable condition on postoperative day 3. At the 30-day follow-up, the patient had an uneventful recovery, and transthoracic echocardiography demonstrated marked improvement in LV function, with the ejection fraction increasing from a preoperative value of 25% to 30% to a postoperative value of 35% to 40%. The MVr remained competent, with no evidence of residual regurgitation or stenosis, and favorable transmitral flow dynamics. The periareolar incision site showed excellent wound healing, with minimal scarring and an aesthetically favorable appearance (Figure 2). Additionally, cardiac computed tomographic angiography confirmed the patency of both grafts with satisfactory anastomotic configuration. Restoration of LV geometry and a well-repaired mitral valve were also observed (Figure 3). The Video illustrates the preoperative imaging, intraoperative surgical steps, and postoperative outcomes in this patient.

**Figure 2:**
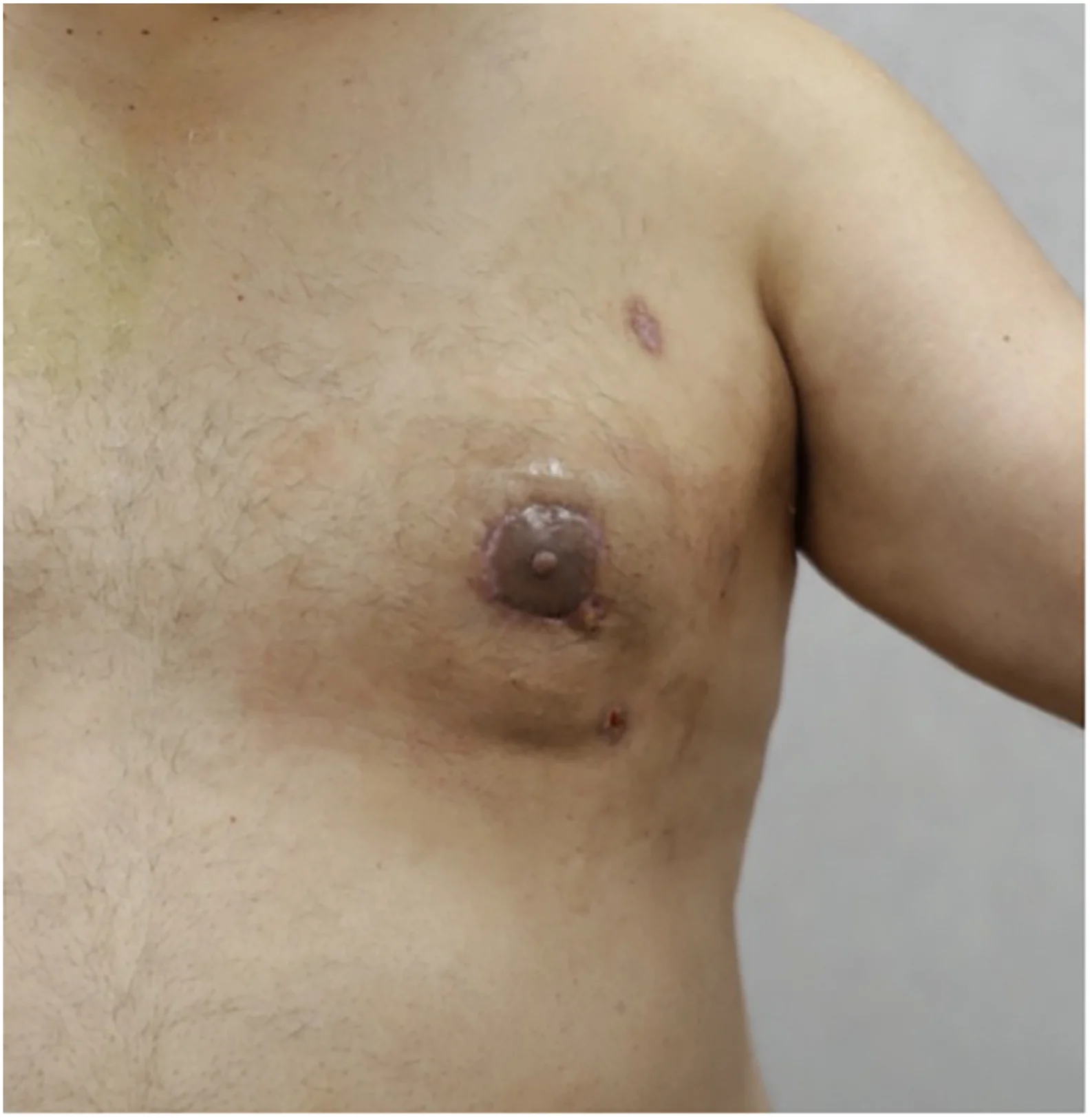
Clinical photograph at 30-day follow-up showing the well-healed left periareolar incision with an excellent cosmetic outcome.

**Figure 3:**
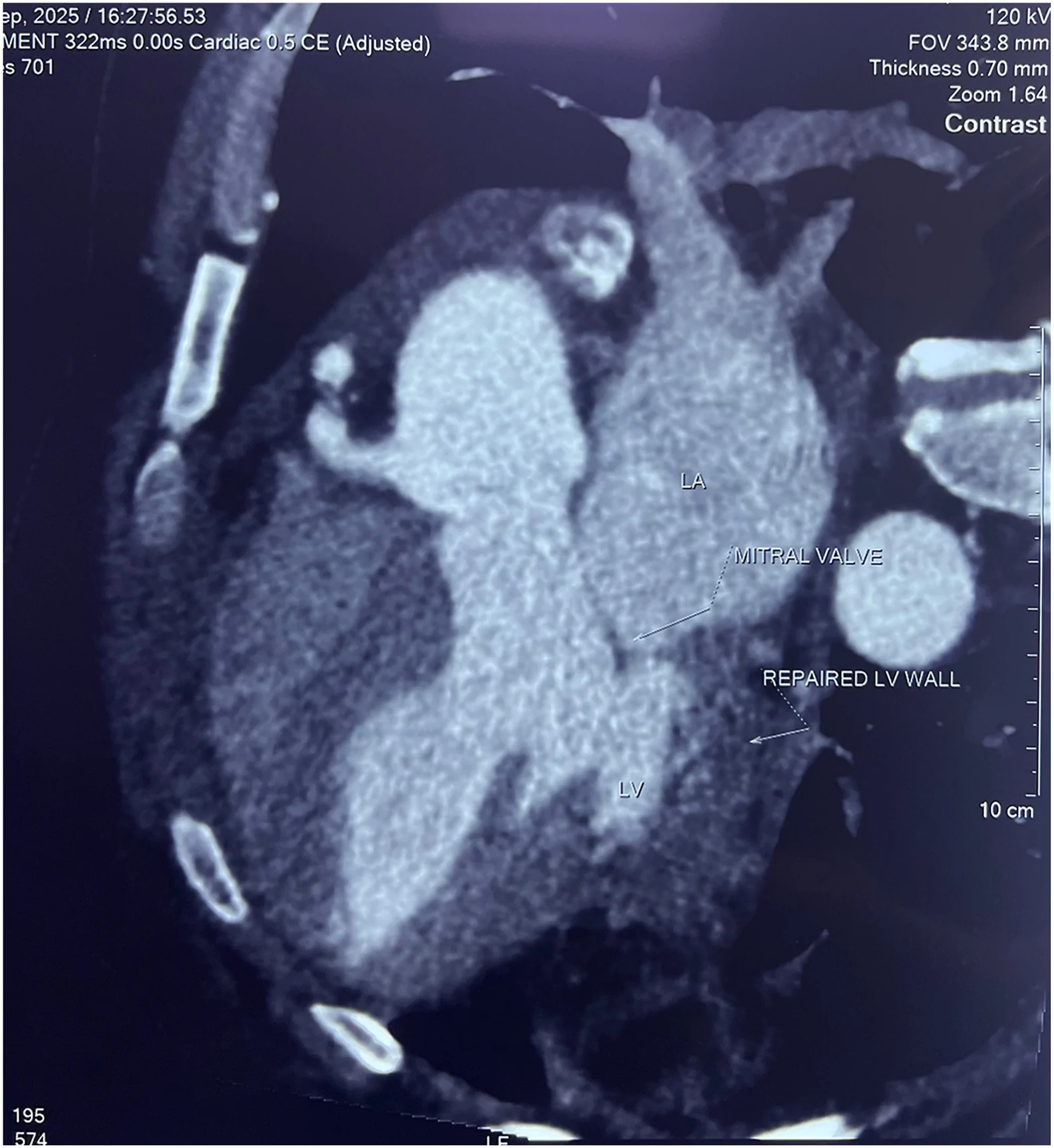
Cardiac computed tomographic angiography showing the repaired mitral valve and restored left ventricular (LV) geometry. (FOV, field of view; LA, left atrium.)

## Comment

This case highlights the practicality and safety of a minimally invasive, endoscopic-assisted periareolar approach for performing triple cardiac surgery—CABG, MVr, and LV pseudoaneurysm repair—with favorable postoperative outcomes and improved cardiac functional stability. Minimally invasive cardiac surgery offers several advantages over conventional full sternotomy, including reduced surgical trauma, lower transfusion requirements, faster recovery, and improved cosmetic outcomes.^5^ Although combined cardiac procedures are traditionally performed through sternotomy, recent advances have shown that multiple interventions can be successfully achieved through a single minimally invasive incision, reducing patient morbidity without compromising surgical efficacy.^6^ Our previous experience at our center (Shar Private Hospital, Erbil, Iraq) with periareolar access for isolated CABG and dual procedures demonstrated excellent outcomes, with minimal postoperative outcomes and high patient satisfaction.^4^ In the present case, partial rib resection was necessary to optimize exposure and facilitate complete pseudoaneurysm repair and secure mitral repair.

In conclusion, periareolar minimally invasive access can be safely extended to complex cardiac procedures. With careful planning and expertise, such procedures can achieve effective outcomes while offering both functional and cosmetic benefits.
